# Evidence of surgical outcomes fluctuates over time: results from a cumulative meta-analysis of laparoscopic versus open appendectomy for acute appendicitis

**DOI:** 10.1186/s12876-016-0453-0

**Published:** 2016-03-15

**Authors:** Tomohiko Ukai, Satoru Shikata, Hiromu Takeda, Lauren Dawes, Yoshinori Noguchi, Takeo Nakayama, Yousuke C. Takemura

**Affiliations:** Department of Community Medicine, Mie University School of Medicine, 2-174 Edobashi, Tsu, Mie 514-8507 Japan; Department of Family Medicine, Mie University School of Medicine & Graduate School of Medicine, 2-174 Edobashi, Tsu, Mie 514-8507 Japan; Department of Family Medicine, Mie Prefectural Ichishi Hospital, 616 Minamiieki, Hakunsan-cho, Tsu, Mie 515-3133 Japan; Department of Surgery, Flinders Medical Centre, Bedford Park, SA 5042 Australia; General Internal Medicine, Japanese Red Cross Nagoya Daini Hospital, 2-9 Myoken-cho, Showa-ku, Nagoya, 466-8650 Aichi Japan; Department of Health Informatics, Kyoto University School of Public Health, Konoe-cho, Yoshida, Sakyo-ku, Kyoto 606-8501 Japan

**Keywords:** Cumulative meta-analysis, Randomized controlled trials, Laparoscopic appendectomy, Open appendectomy

## Abstract

**Background:**

In surgical trials, complex variables such as equipment development and surgeons’ learning curve are involved. The evidence obtained in these trials can thus fluctuate over time. We explored the stability of the evidence obtained during surgery by conducting a cumulative meta-analysis of randomized controlled trials for open and laparoscopic appendectomy.

**Methods:**

We conducted a cumulative meta-analysis of randomized controlled trials comparing laparoscopic appendectomy with open appendectomy for acute appendicitis, a topic with the greatest number of trials in the gastroenterological surgical field. We searched the MEDLINE (PubMed), EMBASE, and CINAHL databases up to September 2014 and reviewed the bibliographies. Outcomes were the incidence of intra-abdominal abscess, incidence of wound infection, operative time, and length of hospital stay. We used the 95 % confidence interval (95 % CI) of effect size for the significance test.

**Results:**

Sixty-four trials were included in this analysis. Of the 51 trials addressing intra-abdominal abscesses, our cumulative meta-analysis of trials published up to and including 2001 demonstrated statistical significance in favor of open appendectomy (cumulative odds ratio [OR] 2.35, 95 % CI 1.30–4.25). The effect size in favor of open procedures began to disappear after 2001, leading to an insignificant result with an overall cumulative OR of 1.32 (95 % CI 0.84–2.10) when laparoscopic appendectomy was compared with open appendectomy.

**Conclusions:**

The evidence regarding treatment effectiveness changed over time, after treatment effectiveness became significant in trials comparing laparoscopic and open appendectomy. Observing only the 95 % confidence interval of effect size from a meta-analysis may not provide conclusive results.

**Electronic supplementary material:**

The online version of this article (doi:10.1186/s12876-016-0453-0) contains supplementary material, which is available to authorized users.

## Background

Meta-analyses of randomized controlled trials (RCTs), which combine the evidence presented in individual research reports, are expected to produce the highest level of evidence and have accordingly become increasingly important in health care [1]. The concept of a cumulative meta-analysis, which is reanalyzed each time the results of a new trial are published, was introduced by Lau et al. in 1992 [2]. This technique was designed to enable determinations of both clinical efficacy and harm as well as the tracking of trials and planning of future trials [3].

The 1992 study by Lau et al. indicated that two very large clinical trials on the efficacy of streptokinase for acute myocardial infarction [4, 5] may have been unnecessary because, according to their cumulative meta-analysis, the treatment efficacy was already statistically significant before those two trials were conducted. Later, cumulative meta-analyses of other topics demonstrated that statistically significant results in meta-analyses can later disappear, especially when well-powered and well-designed trials with sufficient numbers of outcomes and patients appear. In the surgical field, it is quite possible that once a surgical intervention is established, evidence regarding its effectiveness can change over time because of the complexity of surgical trials, which involve advances in surgical equipment and techniques, progress in surgeons’ learning curves as they develop novel skills, and variations in postoperative management, among other factors [6].

To identify changes in the evidence obtained in surgical trials over time, we selected trials comparing the clinical effectiveness of laparoscopic appendectomy and open appendectomy for acute appendicitis. We considered this topic suitable for the observation of chronological trends because, to the best of our knowledge, this topic is associated with the highest number of RCTs in the gastroenterological surgical field [7]. In light of the existing meta-analyses on this topic, including a Cochrane review [8–10], our purpose was to identify any changes in the evidence over time rather than the superiority of one procedure over the other.

We asked the following clinical question: might the evidence demonstrated by a meta-analysis of RCTs of surgical procedures change over time? To answer this question, we conducted a cumulative meta-analysis of RCTs that had compared laparoscopic appendectomy with open appendectomy.

## Methods

Herein we conducted a cumulative meta-analysis of RCTs to ascertain chronological trends in the comparison of laparoscopic appendectomy and open appendectomy for acute appendicitis. We used the cumulative meta-analysis technique introduced by Lau et al. in 1992 [2]. In a cumulative meta-analysis, studies are added one at a time according to their date of publication, and the results are summarized as each new study is added.

### Literature search

We systemically searched the MEDLINE (PubMed), EMBASE, and CINAHL databases for articles in all languages that described RCTs published between 1991, when laparoscopic appendectomy was initiated, and September 2014. In MEDLINE, we utilized the CRD/Cochrane Highly Sensitive Search Strategy [11] with the search terms “appendectomy” and “appendicitis.” We performed the EMBASE search strategy to optimize sensitivity and specificity [12] with the terms “appendectomy” or “appendicitis.” We searched the CINAHL database using a strategy in which terms with the best optimization of sensitivity and specificity [13] were combined with “appendectomy” or “appendicitis.” Reference lists of the review articles and previously published meta-analyses were searched by hand. The search was last done on December 18, 2014.

### Selection criteria for studies in this review

Our inclusion criteria were as follows: (1) prospective RCTs, (2) studies comparing laparoscopic surgery and open surgery for acute appendicitis, (3) studies with human adult participants, and (4) studies written in any language. We excluded studies with any of the following characteristics: (1) pediatric participants, (2) comparisons of diagnostic efficacy, and (3) assessment of the effectiveness of variations of standard laparoscopic techniques, such as the single trocar technique versus the standard technique.

### Outcome measures

Outcomes included the incidence of intra-abdominal abscess, the incidence of wound infection, the operative time, and the length of hospital stay. We adopted these four outcome measures because these are most frequently measured in RCTs addressing this topic.

### Assessment of study quality

We assessed the risk of bias with respect to adequate sequence generation, allocation concealment, blinding, incomplete outcome data addressed, and selective reporting. Two authors (TU and HT) assessed the studies that met the inclusion criteria (Table 1).

**Table 1:** Outcomes and assessment of risk of bias in the included studies

		Outcomes				Risk of bias				
Author	Year	Wound infection	Abscess	Operative time	Length of hospital stay	Adequate sequence generation?	Allocation concealment?	Blinding?	Incomplete outcome data addressed?	Free of selective reporting?
Al-Mulhim	2002	○	○	–	–	?	+	?	?	?
Attwood	1992	○	○	–	–	?	+	?	+	?
Barth	1999	○	–	–	–	?	?	?	?	?
Bauwens	1999	○	○	○	○	?	+	?	-	+
Bruwer	2000	○	○	○	○	+	+	?	+	?
Cox	1996	○	○	○	○	?	?	?	?	?
Eichen	1994	○	○	○	○	+	+	?	?	?
Frazee	1994	○	○	○	○	?	?	?	+	?
Gouder	2011	○	–	–	○	?	?	?	-	?
Hall Long	2000	○	○	–	○	?	+	?	?	?
Hansen	1996	○	○	–	–	+	+	?	-	?
Hart	1996	○	○	○	○	+	+	?	-	+
Hebebrand	1994	○	○	○	○	?	+	-	+	+
Heikkinen	1998	○	○	–	–	?	+	?	?	?
Hellberg	1999	○	○	–	–	+	+	?	-	?
Helmy	2001	–	○	–	–	?	?	?	?	?
Henle	1996	○	○	–	–	+	?	?	-	?
Huang	2001	○	○	○	○	?	+	+	?	?
Ignacio	2003	○	○	○	○	+	+	+	+	?
Jan	2011	○	○	○	–	+	?	?	+	?
Kaiser	2006	○	○	○	○	?	+	?	+	+
Kald	1999	○	○	–	–	+	+	?	-	+
Kaplan	2009	○	–	○	○	?	?	?	+	?
Karadayi	2003	○	○	○	–	+	?	?	-	+
Kargar	2010	○	–	○	○	+	+	?	?	?
Katkhouda	2005	○	○	–	–	+	+	+	-	+
Kazemier	1997	○	○	○	○	+	+	?	+	?
Kehagias	2009	○	○	–	–	+	+	?	?	?
Khalil	2011	○	–	○	○	+	?	?	-	?
Kocatas	2013	○	○	–	○	+	+	?	-	?
Kouhia	2010	○	○	–	–	?	+	?	+	+
Kum	1993	○	○	○	○	+	+	?	-	?
Laine	1997	○	○	○	○	?	?	?	+	?
Macarulla	1995	○	○	○	○	?	+	?	+	?
Martin	1995	○	○	○	○	?	?	?	+	?
Minne	1997	○	○	–	–	+	?	?	-	?
Moberg	2005	○	○	–	–	+	+	+	+	+
Moirangthem	2008	○	–	○	○	?	?	?	+	?
Mutter	1996	○	○	○	–	?	?	?	+	?
Navarra	2000	○	○	○	○	+	+	?	+	?
Nordentoft	2000	–	–	○	–	?	?	?	-	?
Olmi	2005	○	○	–	–	?	?	?	?	?
Ortega	1996	○	○	○	○	+	+	+	+	?
Özmen	1999	○	○	○	○	?	?	?	?	?
Pedersen	2001	○	○	–	–	+	+	?	?	+
Pozo	1996	○	○	○	○	?	?	?	+	?
Rashid	2013	○	–	○	○	+	?	?	+	?
Reiertsen	1997	○	○	○	○	?	?	-	-	?
Ricca	2007	○	○	○	–	+	+	+	+	?
Schippers	1997	○	○	○	–	?	+	?	+	?
Settmacher	1995	–	–	○	–	?	+	?	?	+
Sezeur	1997	○	○	○	○	?	+	?	-	?
Shirazi	2010	○	–	○	○	?	?	?	?	?
Stare	1998	○	○	○	–	?	+	-	+	?
Sun	1998	○	○	○	○	?	?	?	+	?
Tate	1993	○	○	○	○	+	+	?	+	?
Tzovaras	2010	○	–	–	–	+	+	?	+	?
Vallribera	2003	○	○	○	○	+	+	?	+	?
van Dalen	2003	–	–	○	–	?	+	?	+	?
Wei	2010	○	○	○	○	?	?	?	+	?
Williams	1996	○	○	○	○	?	?	?	-	?
Witten	1998	○	○	○	○	+	+	-	+	?
Yin	1996	○	–	–	○	+	+	+	+	?
Zhang	1998	○	○	○	○	+	?	?	?	?

+, low risk of bias; −, high risk of bias; ?, unclear risk of bias

### Data extraction

Binary data were extracted for the incidences of intra-abdominal abscess and wound infection, and continuous data were extracted for the operative time and length of hospital stay. Two authors (TU and HT) independently undertook this process, and disagreements were resolved through discussion. Participants who were converted intraoperatively from laparoscopic appendectomy to open appendectomy were included in the laparoscopic appendectomy arm on an intention-to-treat basis.

### Statistical analysis

A meta-analysis was performed using Review Manager (RevMan) software, version 5.3.5, provided by the Cochrane Collaboration, Copenhagen, Denmark. Since a cumulative meta-analysis cannot be performed by RevMan, we used Comprehensive Meta Analysis software, version 3.3.070 (Biostat, Englewood NJ, USA). For the binary variables (i.e., the incidence of intra-abdominal abscess and wound infection), the statistical analyses were performed using the odds ratio (OR) of laparoscopic appendectomy to open appendectomy as the summary statistic. The OR point estimate was considered significant at the *p* < 0.05 level when the 95 % confidence interval (95 % CI) did not include the value 1. For the continuous variables (i.e., the operative time and the length of hospital stay), the statistical analyses were performed using the mean difference (MD) as the summary statistic, i.e., the time taken for an laparoscopic appendectomy subtracted by the time taken for an open appendectomy. The MD point estimate was considered significant at the *p* < 0.05 level if the 95 % CI did not include the value 0.

We used both the fixed-effects model and the random-effects model according to the Mantel-Haenszel method [14] for the statistical analysis. The fixed-effects model assumes the homogeneity of the true treatment effect, whereas the random-effects model accepts between-study differences in the treatment effects. The confidence interval thus tends to be wider in the random-effects model when a certain level of treatment effect heterogeneity is observed. We performed both the fixed-effects model and random-effects model, and if their results were similar, the random-effects model was adopted.

We also tested for study homogeneity by calculating *I*^2^. This value can be calculated as *I*^2^ = 100 % × (Q ‐ df)/Q, where Q is Cochran’s heterogeneity statistic and df is the degree of freedom [15]. An outcome with no events was considered a “zero cell” in the 2 × 2 table. Although correction is needed to pool the ORs of studies that include zero cells, this can influence the results and possibly introduce bias [16]. To conduct a bias-free meta-analysis, we used the Mantel-Haenszel model with a correction factor of 0.5 (0.5 was added to all cells in the 2 × 2 table when there was a zero cell).

## Results

**Fig. 1:**
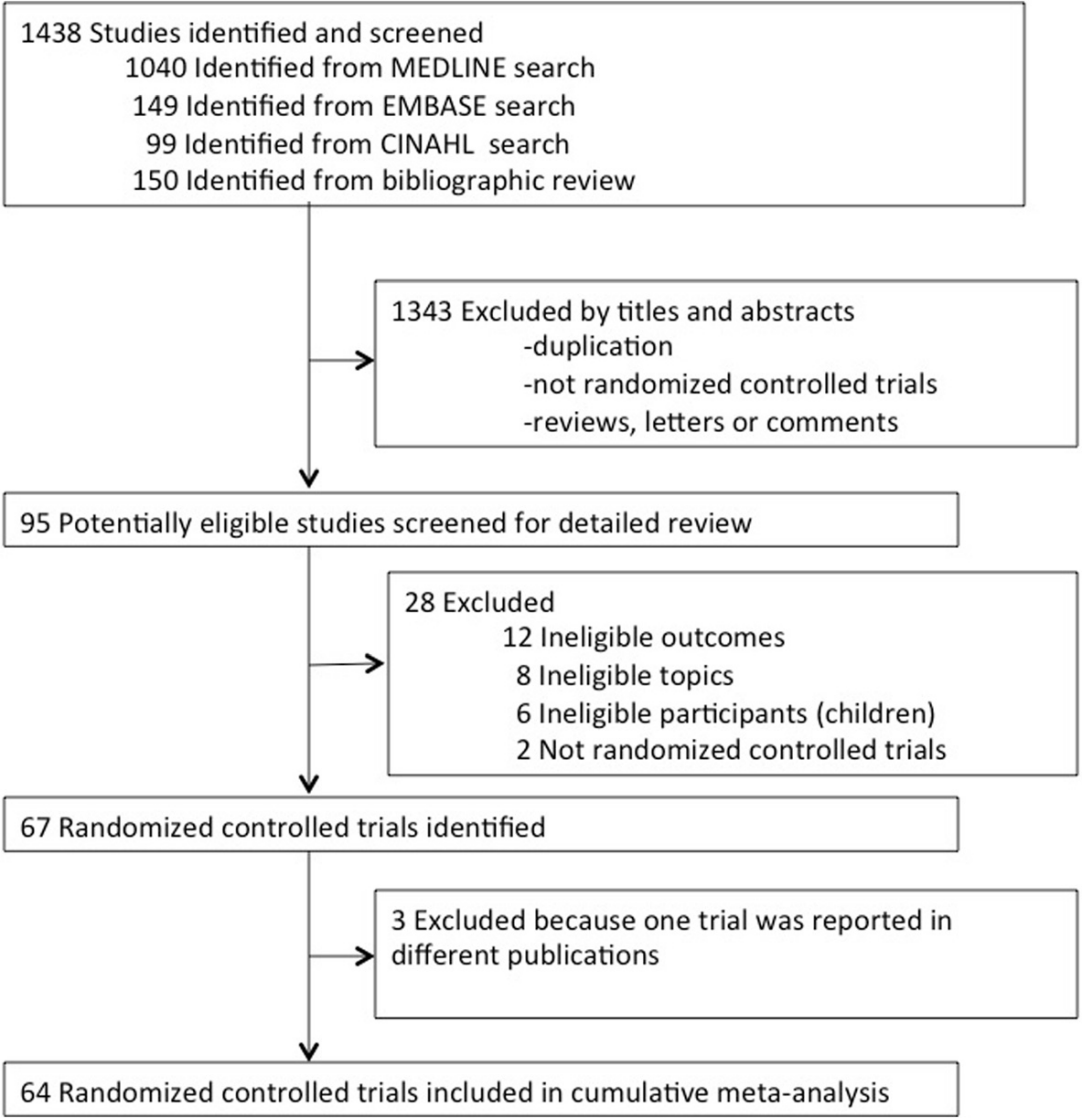
Flowchart of trial identification for cumulative meta-analysis

Our database and bibliography searches yielded 1,438 and 150 articles. After eliminating duplicate articles, we evaluated the titles and abstracts of these studies according to the inclusion and exclusion criteria, after which 95 articles remained (Fig. 1). After the full texts of these articles were read and the ineligible studies were excluded, 64 RCTs published from 1992 to 2012 were used for the data extraction (Table 1).

### Intra-abdominal abscess

**Fig. 2:**
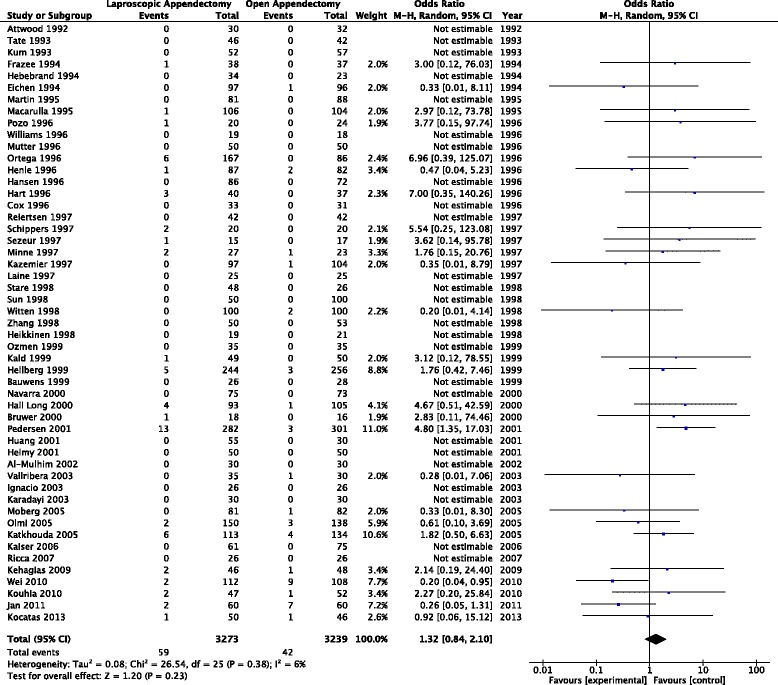
Pooled odds ratio in intra-abdominal abscess for trials comparing laparoscopic appendectomy and open appendectomy

**Fig. 3:**
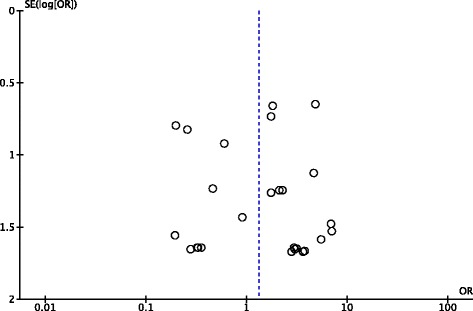
Funnel plot of trials comparing laparoscopic appendectomy and open appendectomy for intra-abdominal abscess

**Fig. 4:**
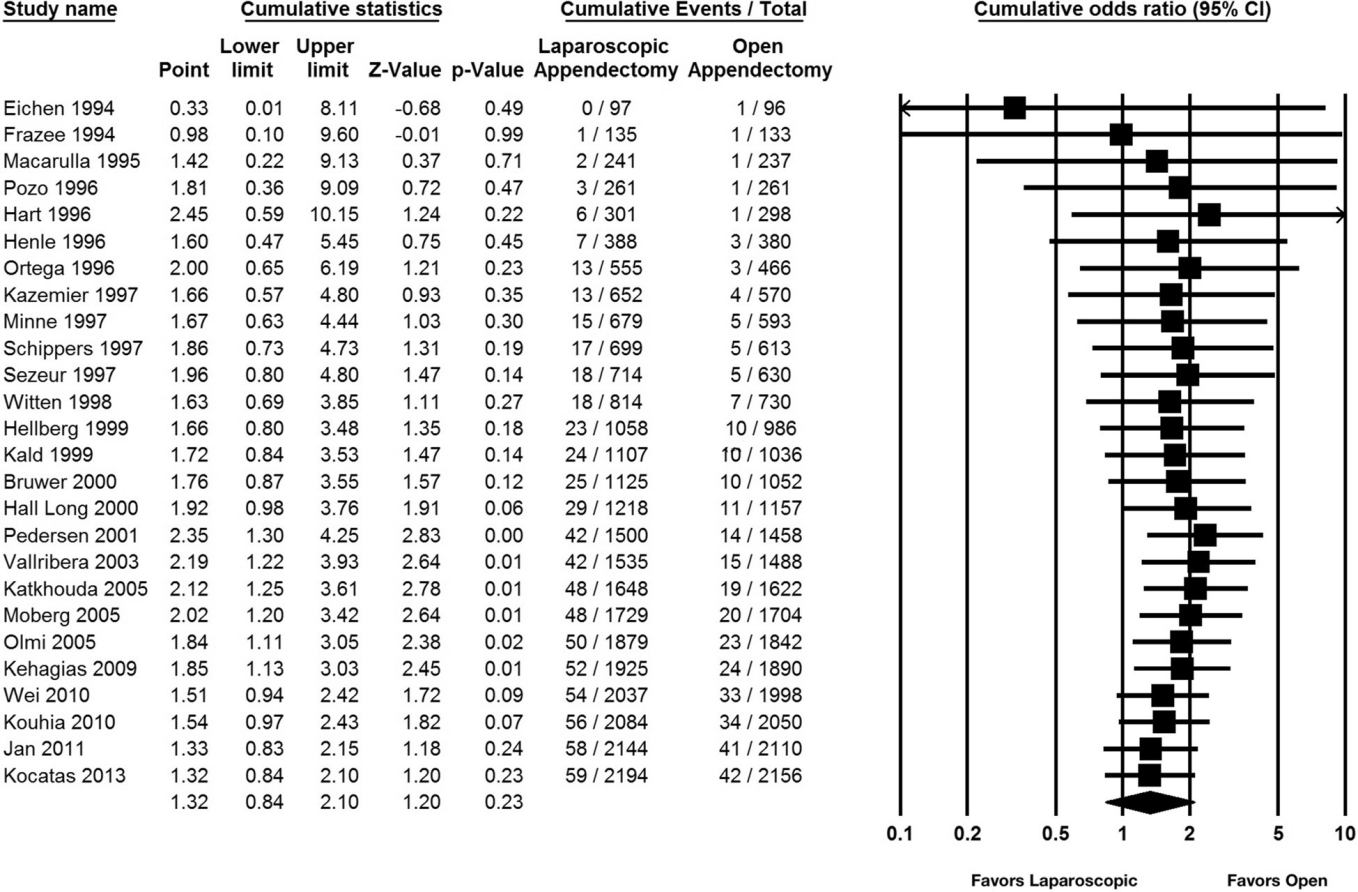
Cumulative odds ratio in intra-abdominal abscess comparing laparoscopic appendectomy and open appendectomy. Studies with zero event in both laparoscopic and open appendectomy are not included

This outcome analysis included 51 relevant studies with a total of 6,512 participants (3,273 for laparoscopic appendectomy and 3,239 for open appendectomy) (Fig. 2). The total numbers of events were 61 in the laparoscopic appendectomy group (1.80 %) and 43 in the open appendectomy group (1.30 %). The overall OR was 1.34 (95 % CI 0.92–1.94) in the fixed-effects model and 1.32 (95 % CI 0.84–2.10) in the random-effects model. The overall *I*^*2*^ was 6 %. A visual inspection of the funnel plot for small-study effects did not show asymmetry (Fig. 3). A cumulative meta-analysis demonstrated that the CI narrowed until it identified the first significant difference in favor of open appendectomy in the trial published in 2001 (OR 2.35, 95 % CI 1.30–4.25). However, as more studies were added, the CI shifted to the left in favor of laparoscopic appendectomy. Finally, the CI included the value 1 in 2010, and there was no significant difference (Fig. 4).

### Wound infection

**Fig. 5:**
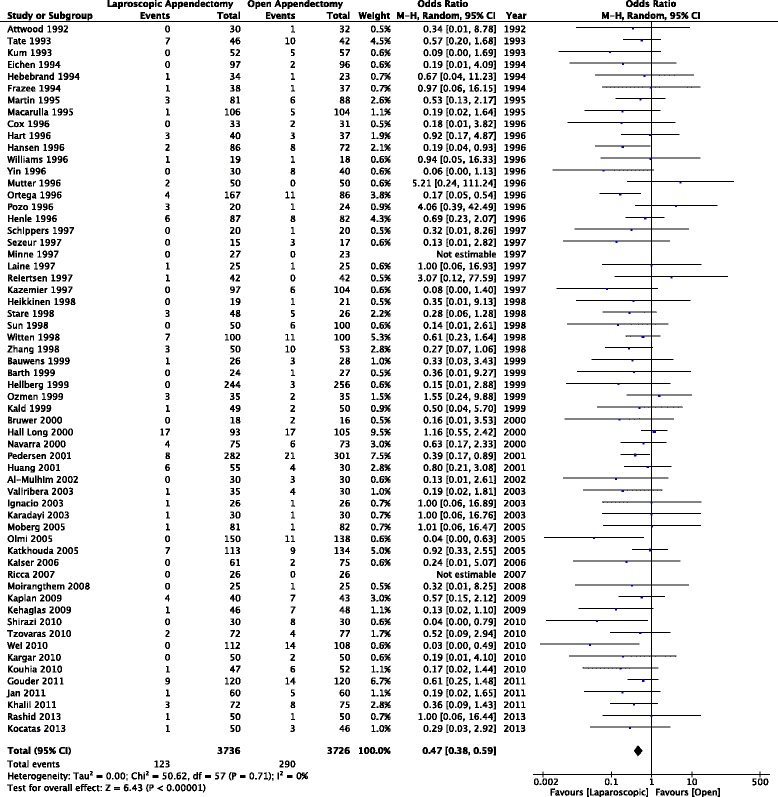
Pooled odds ratio in wound infection for trials comparing laparoscopic appendectomy and open appendectomy

**Fig. 6:**
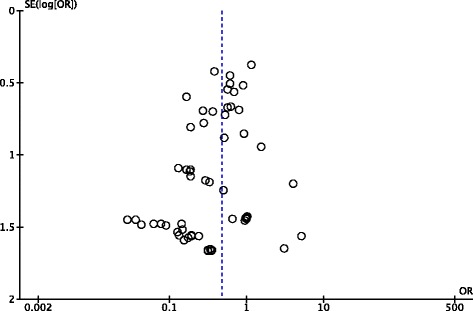
Funnel plot of trials comparing laparoscopic appendectomy and open appendectomy for wound infection. OR, odds ratio; SE, standard error

**Fig. 7:**
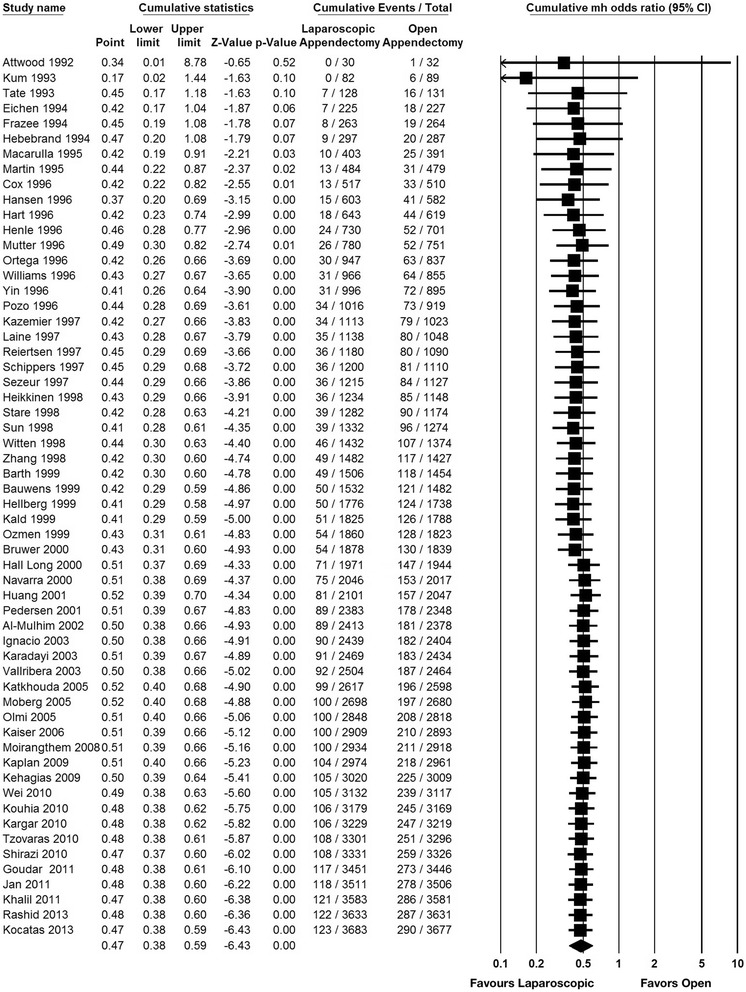
Cumulative odds ratio in wound infection comparing laparoscopic appendectomy and open appendectomy. Studies with zero event in both laparoscopic and open appendectomy are not included

Sixty studies and 7,462 participants (3,736 for laparoscopic appendectomy and 3,726 for open appendectomy) were included for this outcome (Fig. 5). The total numbers of events were 123 in the laparoscopic appendectomy group (3.29 %) and 290 in the open appendectomy group (7.78 %). The overall OR was 0.41 (95 % CI 0.33–0.51) in the fixed-effects model and 0.47 (95 % CI 0.38–0.59) in the random-effects model. The overall *I*^*2*^ was 0 %. A visual inspection of the funnel plot showed slight asymmetry in small studies (Fig. 6). A cumulative meta-analysis showed that the significant difference was first observed in the seventh study in 1995, and that this trend did not change substantially with subsequent studies (Fig. 7).

### Operative time

There were 43 studies with a total of 4,202 participants (2,135 for laparoscopic appendectomy and 2,067 for open appendectomy) that compared the operative time between laparoscopic appendectomy and open appendectomy (Fig. 4). The average operative times were 57.3 min for laparoscopic appendectomy and 47.0 min for open appendectomy. The overall MD was 4.4 min longer for laparoscopic appendectomy (95 % CI 3.5–5.3) in the fixed-effects model and 10.1 min (95 % CI 5.9–14.3) in the random-effects model. The *I*^*2*^ value was 95 %. Significant heterogeneity was found, and thus a cumulative meta-analysis was not performed.

### Length of hospital stay

Thirty-nine studies with a total of 4,240 participants (2,165 for laparoscopic appendectomy and 2,153 for open appendectomy) were included for this outcome (Fig. 5). The average length of hospital stay was 3.21 days for laparoscopic appendectomy and 4.40 days for open appendectomy. The MD of the length of hospital stay was 1.08 days shorter for laparoscopic appendectomy (95 % CI 1.01–1.75) in the fixed-effects model and 1.12 days (95 % CI 0.77–1.47) for the random-effects model. The *I*^*2*^ value was 95 %. Because significant heterogeneity was found, a cumulative meta-analysis was not performed.

## Discussion

Our cumulative meta-analysis of the comparison of laparoscopic appendectomy and open appendectomy for acute appendicitis demonstrated that the evidence provided by the meta-analysis of surgical RCTs can change over time. Intra-abdominal abscesses were significantly more frequent in the laparoscopic appendectomy group during the period from 2001 to 2009, but this significance disappeared as more trials accumulated. Our present findings visually demonstrated how evidence changes over time in the surgical field. Although other outcome measures did not exhibit the same transition as intra-abdominal abscess, all of the outcome measures demonstrated similar trends in favor of laparoscopic appendectomy.

### Fluctuation of evidence

When there is evidence concerning the effectiveness of a medical intervention, one can reasonably conclude that no further research is needed on the topic. However, previous studies have shown that the results of meta-analyses are underused, and many RCTs are conducted even after significant evidence has been demonstrated through a meta-analysis [2, 17]. Some researchers have contended that it is unethical and a waste of resources to randomize participants in unnecessary trials, and they emphasized the importance of avoiding redundant trials. In the meta-analysis from a Cochrane review published in 2010 [10], intra-abdominal abscess was significantly more frequent in laparoscopic appendectomy than open appendectomy (albeit with moderate heterogeneity), but the present study illustrates that a significant result turned insignificant. The findings of our study thus provide an example in which large intervention effects are not always conclusive, and they fluctuate over time. Fluctuation was not found in the wound infection outcome data, and the result was consistently in favor of laparoscopic appendectomy. Penninga et al. have shown strong evidence of favoring laparoscopic appendectomy for wound infection using a trial sequential analysis [18], and our result is consistent with this.

### The nature of surgical trials

Evidential instability can be explained by the nature of surgical interventions, which are highly complex and difficult to evaluate [6]. Surgical interventions involve many factors, including the surgeons’ skill and judgment, the skills of the treating team, the development of surgical devices, and pre- and post-surgical management. All of these factors change on a daily basis. Second, the effect of the learning curve influences outcomes [19]. For example, surgeons’ performances improve to the point of acquiring expertise as they gain training and experience. The observed shift toward favoring laparoscopic appendectomy, which we observed in later trials, might be explained by the effects of these factors. Because of this phenomenon, surgical trials may differ from pharmaceutical trials, as in the latter, theoretically efficacy does not change over time. To minimize these factors, trial designs that consider the effects of the learning curve or perioperative management should be used [20, 21].

### The shift favoring conservative treatment in the early to middle period

Although we observed a shift favoring laparoscopic appendectomy in later trials in our analysis, an apparent shift in the opposite direction was observed in the early to middle period after good results were obtained for laparoscopic appendectomy in very early trials. Early trials tend to overestimate treatment effects for a variety of reasons, such as the under-reporting of disappointing results or the selection of favorable subgroups [22–24]. However, as new interventions are disseminated and the study participant inclusion criteria are broadened, positive results become less extreme. Relevant examples can be found elsewhere [25, 26]. We assume that the results favoring open appendectomy in the early-middle period are another example of this phenomenon.

### Limitations

This study has several limitations. First, we observed the change from statistically significant to insignificant findings using the 95 % CI of odds ratios. There are other methods to analyze the results of meta-analyses chronologically, such as the trial sequential analysis (TSA) which takes into account random errors due to repetitive meta-analyses [27, 28]. We performed a TSA for the intra-abdominal abscess outcome, and it did not show significance throughout; i.e., the required information size was not reached and the Z-curve did not cross the trial sequential monitoring boundaries. Therefore, we cannot dismiss the possibility of random errors which brought the statistical significance in the analysis. This strengthens the importance of not relying only on the 95 % CI of the effect size and of performing a TSA to conclude the comparison.

Second, this analysis can be prone to publication bias. It is likely that trials with contradicting results would be published more often than those confirming the existing evidence. Small study effects could also have existed in our analysis. A visual inspection of the funnel plot showed slight asymmetry in small studies (Fig. 6).

Third, we did not conduct an analysis of the operative time or the length of hospital stay due to considerable heterogeneity, and a small study effect could have a substantial impact on the heterogeneity.

Fourth, studies with a high risk of bias could skew the results. We conducted subgroup analyses for trials with low and high risks of bias, and the results showed that the intra-abdominal abscess outcome after laparoscopic appendectomy was considerably more frequent among the studies with a low risk of bias compared to those with a high risk of bias, although a cumulative meta-analysis showed a similar trend toward favoring laparoscopic appendectomy [see the Additional file 1].

Fifth, the rarity of intra-abdominal abscesses may have complicated the analysis. The small number of events can increase the uncertainty. Since there are many trials with zero-events in intra-abdominal abscess, we substituted the correction factor of 0.5 to 0.01 as a sensitivity analysis to test for robustness [29]. The cumulative meta-analysis showed that statistical significance was first observed in the trial published in 2001 and it disappeared as more results accumulated, with the overall OR of 1.24 (95 % CI 0.84–1.81). The results were similar between both correction factors.

Sixth, the number of studies published each year was unbalanced. We included more trials during the time period from 1996 to 2001, which might have biased the results. Nevertheless, considering that the shift from surgeons favoring open appendectomy to those favoring laparoscopic appendectomy occurred after 2002, new findings might have been more evident if more trials had been published after 2001.

Finally, although we report herein an example demonstrating evidential instability in the surgical field, we cannot generalize this observation to all surgical interventions or other fields. Although the numbers of RCTs addressing other surgical topics are generally low [30], more evidence should be accumulated for other topics to understand the stability of evidence by means of not only cumulative meta-analyses, but also TSAs.

## Conclusion

Our cumulative meta-analysis of RCTs comparing laparoscopic and open appendectomy demonstrated that evidence can fluctuate over time in surgery with complex variables. Observing only the 95 % confidence interval of the effect size from meta-analyses may not provide conclusive results. More stringent analyses should be used to assess the results of meta-analyses.

## Additional file

Additional file 1: Pooled odds ratio in intra-abdominal abscess for trials comparing laparoscopic appendcetomy and open appendectomy among studies with low risk of bias. Cummulative odds ratio in intra-abdominal abscess comparing laparoscopic appendectomy and open appendectomy among studies with low risk of bias. Pooled odds ratio in intra-abdominal abscess for trials comparing laparoscopic appendcetomy and open appendectomy among studies with high risk of bias. Cummulative odds ratio in intra-abdominal abscess comparing laparoscopic appendectomy and open appendectomy among studies with high risk of bias. (ZIP 1902 kb)

## Abbreviations

MD: mean difference
RCT: randomized controlled trials
TSA: trial sequential analysis
