# Supplementary figures and images for: Evidence of surgical outcomes fluctuates over time: results from a cumulative meta-analysis of laparoscopic versus open appendectomy for acute appendicitis
Source: BMC Gastroenterol. 2016 Mar 15;16:37. doi: 10.1186/s12876-016-0453-0 (PMC4793521; doi:10.1186/s12876-016-0453-0)

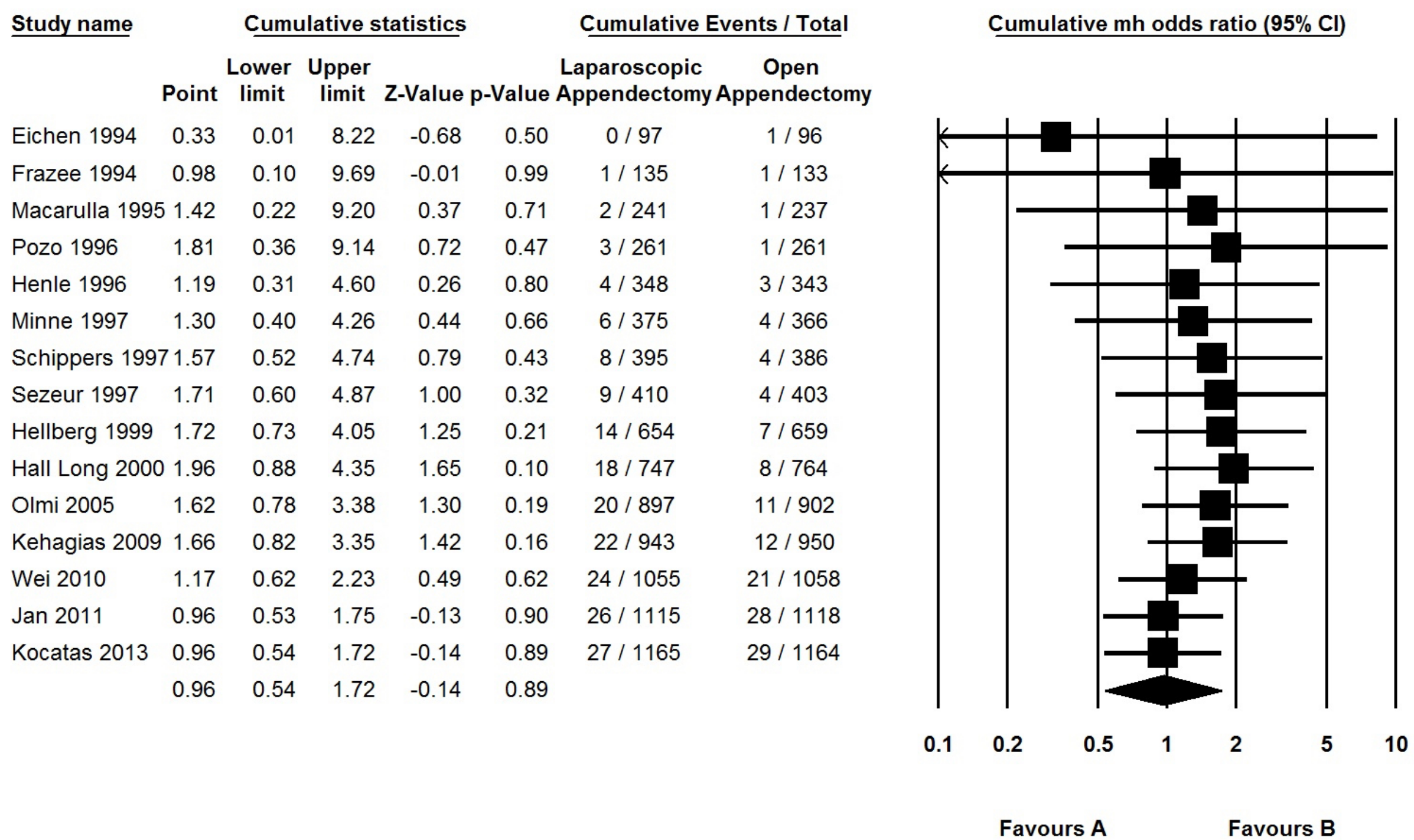

Supplement: Additional file 1: — Pooled odds ratio in intra-abdominal abscess for trials comparing laparoscopic appendcetomy and open appendectomy among studies with low risk of bias. Cummulative odds ratio in intra-abdominal abscess comparing laparoscopic appendectomy and open appendectomy among studies with low risk of bias. Pooled odds ratio in intra-abdominal abscess for trials comparing laparoscopic appendcetomy and open appendectomy among studies with high risk of bias. Cummulative odds ratio in intra-abdominal abscess comparing laparoscopic appendectomy and open appendectomy among studies with high risk of bias. (ZIP 1902 kb) [file 12876_2016_453_MOESM1_ESM.zip › abscess_high bias_R2.pdf]

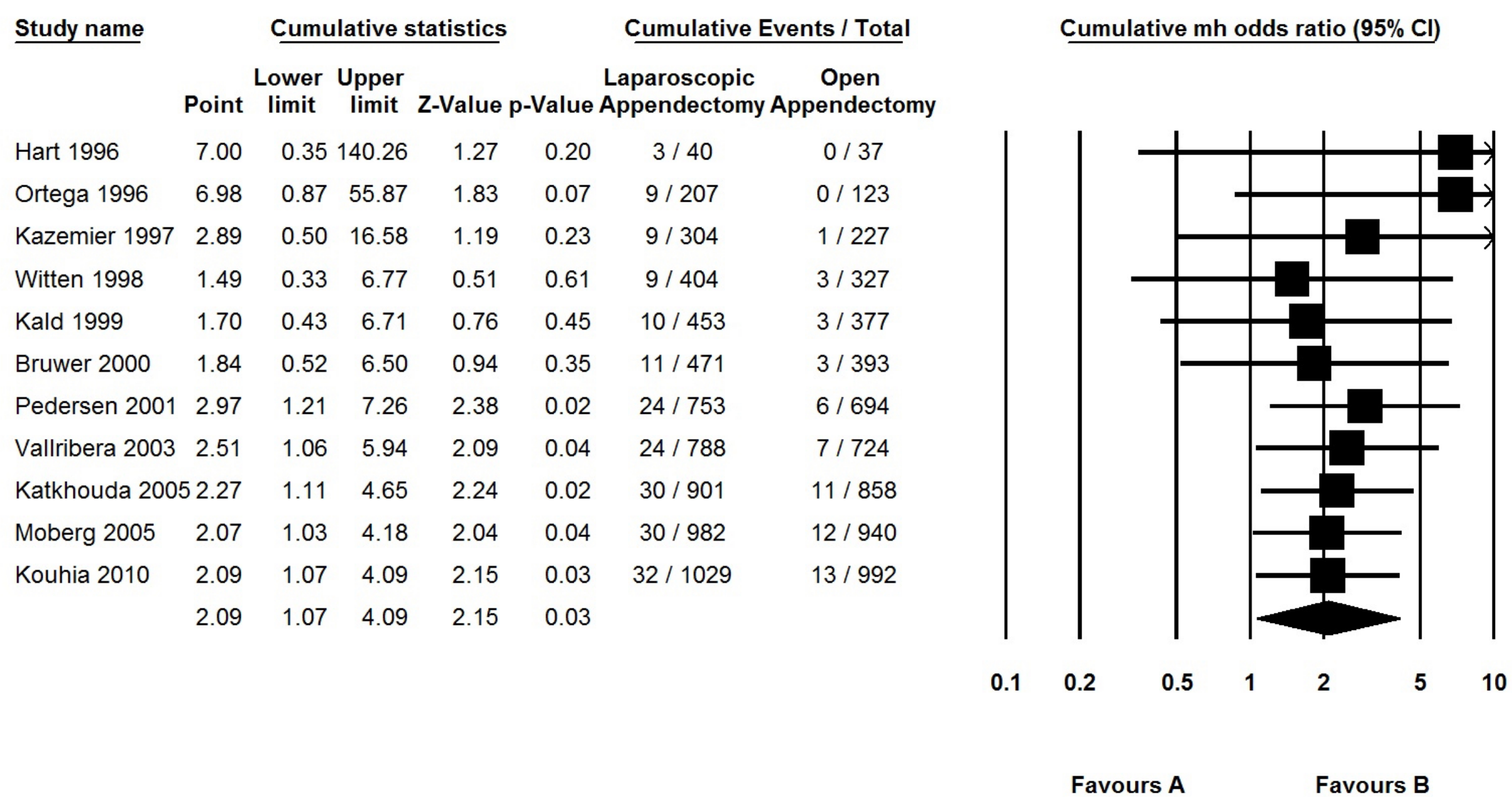

Supplement: Additional file 1: — Pooled odds ratio in intra-abdominal abscess for trials comparing laparoscopic appendcetomy and open appendectomy among studies with low risk of bias. Cummulative odds ratio in intra-abdominal abscess comparing laparoscopic appendectomy and open appendectomy among studies with low risk of bias. Pooled odds ratio in intra-abdominal abscess for trials comparing laparoscopic appendcetomy and open appendectomy among studies with high risk of bias. Cummulative odds ratio in intra-abdominal abscess comparing laparoscopic appendectomy and open appendectomy among studies with high risk of bias. (ZIP 1902 kb) [file 12876_2016_453_MOESM1_ESM.zip › abscess_low risk_no2R2.pdf]

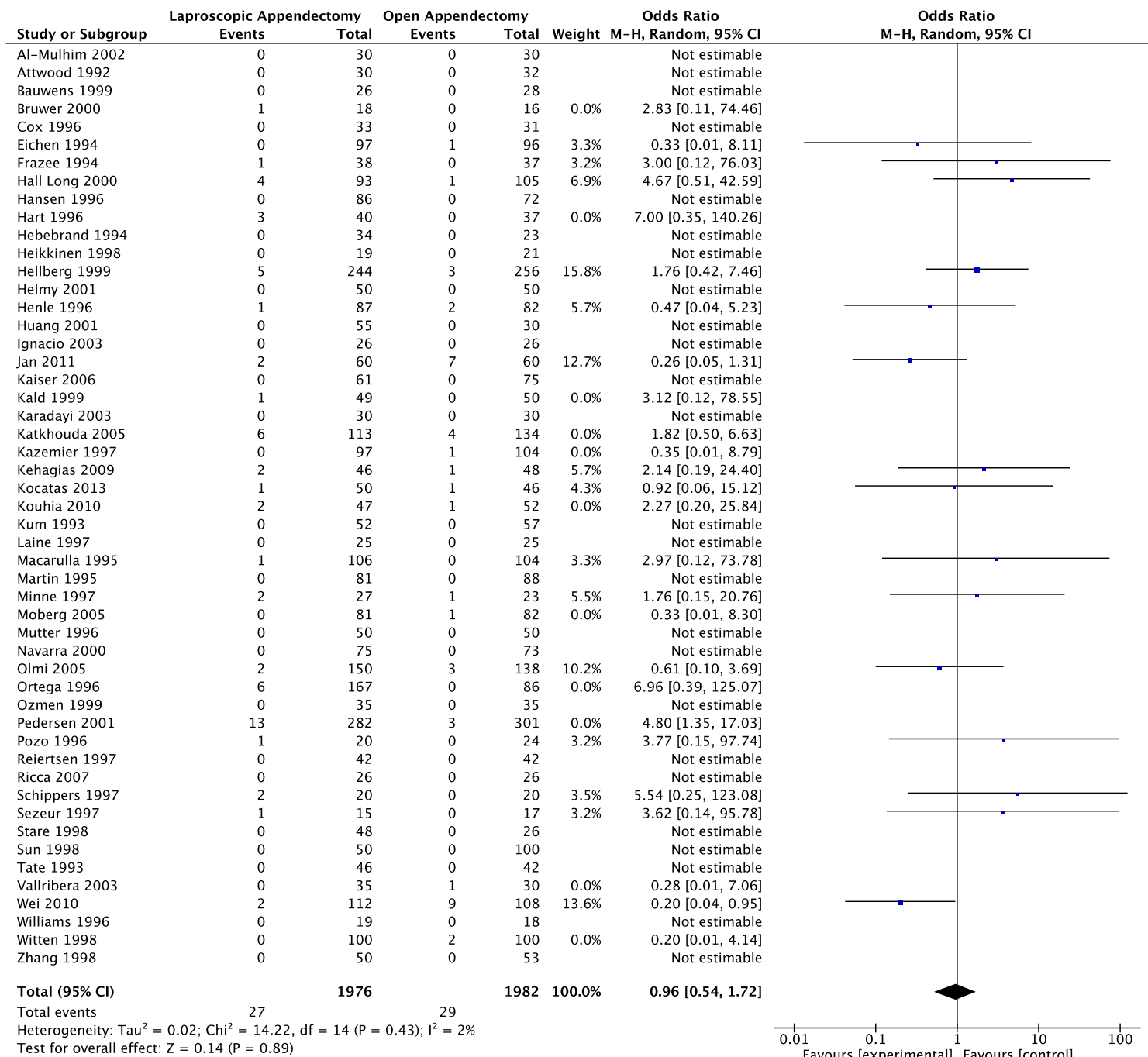

Supplement: Additional file 1: — Pooled odds ratio in intra-abdominal abscess for trials comparing laparoscopic appendcetomy and open appendectomy among studies with low risk of bias. Cummulative odds ratio in intra-abdominal abscess comparing laparoscopic appendectomy and open appendectomy among studies with low risk of bias. Pooled odds ratio in intra-abdominal abscess for trials comparing laparoscopic appendcetomy and open appendectomy among studies with high risk of bias. Cummulative odds ratio in intra-abdominal abscess comparing laparoscopic appendectomy and open appendectomy among studies with high risk of bias. (ZIP 1902 kb) [file 12876_2016_453_MOESM1_ESM.zip › Forest plot_high biasR2.pdf]

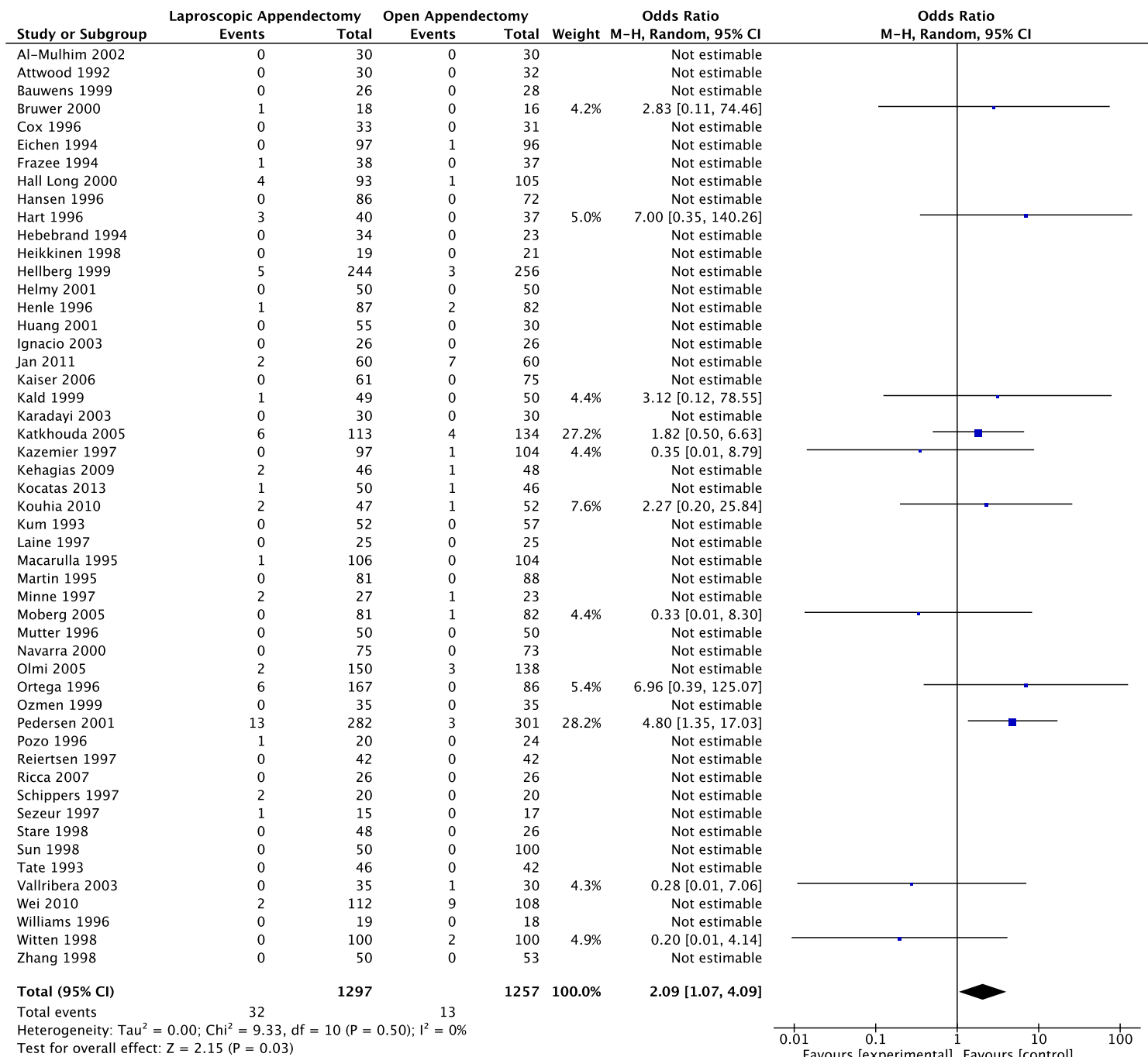

Supplement: Additional file 1: — Pooled odds ratio in intra-abdominal abscess for trials comparing laparoscopic appendcetomy and open appendectomy among studies with low risk of bias. Cummulative odds ratio in intra-abdominal abscess comparing laparoscopic appendectomy and open appendectomy among studies with low risk of bias. Pooled odds ratio in intra-abdominal abscess for trials comparing laparoscopic appendcetomy and open appendectomy among studies with high risk of bias. Cummulative odds ratio in intra-abdominal abscess comparing laparoscopic appendectomy and open appendectomy among studies with high risk of bias. (ZIP 1902 kb) [file 12876_2016_453_MOESM1_ESM.zip › Forest plot_low biasR2.pdf]
